# Plexin domain containing 2 (*PLXDC2*) gene polymorphism rs7081455 may not influence POAG risk in a Saudi cohort

**DOI:** 10.1186/s13104-018-3848-x

**Published:** 2018-10-16

**Authors:** Altaf A. Kondkar, Tahira Sultan, Faisal A. Almobarak, Hatem Kalantan, Khaled K. Abu-Amero, Saleh A. Al-Obeidan

**Affiliations:** Glaucoma Research Chair, Department of Ophthalmology, College of Medicine, King Saud University, P.O. Box 245, Riyadh, 11411 Saudi Arabia

**Keywords:** Genetics, Glaucoma, Middle-east, POAG, rs7081455, Saudi, *PLXDC2*

## Abstract

**Objective:**

Plexin domain containing 2 (PLXDC2), a cell surface transmembrane protein receptor for pigment epithelium derived factor, is expressed in many tissues including the eye. Polymorphism rs7081455 flanking *PLXDC2* has been associated with primary open angle glaucoma (POAG) and its clinical phenotypes and may have a role in POAG. Rs7081455 was genotyped in POAG cases (n = 188) and non-glaucomatous controls (n = 164) of Saudi origin using Taq-Man^®^ to determine any association of this variant with POAG and its endophenotypes.

**Results:**

The risk variant, ‘G’ allele, frequency was 0.56 and 0.52 in controls and POAG cases, respectively (*p *= 0.197) with was no significant deviation from Hardy–Weinberg equilibrium. Genotype analysis between cases and controls revealed no significant distribution under additive (*p *= 0.482), dominant (*p *= 0.590) and recessive models (*p *= 0.228). In addition, glaucoma specific phenotypic traits such as intraocular pressure (IOP) and cup/disc ratio; and number of anti-glaucoma medications, used to assess severity of the disease, were also statistically non-significant. Furthermore, regression analysis showed no significant effect of age, sex and genotype on disease outcome. Rs7081455 was not associated with POAG or its clinical phenotypes such as IOP and cup/disc ratio and hence may not be a significant risk factor for POAG patients of Saudi origin.

**Electronic supplementary material:**

The online version of this article (10.1186/s13104-018-3848-x) contains supplementary material, which is available to authorized users.

## Introduction

Primary open angle glaucoma (POAG) is a chronic optic neuropathy clinically characterized by an open anterior chamber angle. It is the second most common type of glaucoma seen in the Saudi Arabian population [1]. The disease is largely polygenic in nature that follows a genetically complex and multifactorial inheritance pattern leading to blindness [2]. However, the actual pathophysiological and molecular mechanism(s) leading to glaucoma and its progression still remains elusive. Given the complexity and mutational genetic heterogeneity, POAG shows an estimated heritability of 0.81 [3]. Genetic case–control association studies are an important tool to identify genetic features, each of which may show a relatively small effect but may exhibit significant contribution to a large number of cases. Using similar approaches based on candidate gene and genome wide association studies (GWAS), several investigators have identified a number of genetic variants in multiple loci/genes in various ethnic groups, that have been associated with POAG and related inheritable quantitative traits such as intraocular pressure (IOP) and cup/disc ratio (among others) that may have a causal effect on the development and/or progression of the disease [4].

Likewise, Nakano et al. [5] performed a two-stage GWAS in the Japanese population, and demonstrated an association of POAG with genetic variants rs7081455 flanking the plexin domain containing 2 (*PLXDC2*) gene at chromosomal loci 10p12.31. This association was replicated by Mabuchi et al. [6] in another Japanese cohort and demonstrated to influence the risk of POAG by increasing IOP. There is no evidence of any association of this gene and/or variant rs7081455 in the POAG patients of Saudi origin, so we conducted a study to determine whether variant rs7081455 near *PLXD2* is associated with the disease or any of its clinical phenotypes such as IOP or cup/disc ratio and could be a risk factor for POAG in this population.

## Main text

### Methods

#### Settings and study groups

A case–control association study was performed in accordance to the Declaration of Helsinki principles and was approved by the institutional research ethics committee of College of Medicine at the King Saud University (Approval Number # 08-657). All the participants had signed a written informed consent. POAG patients and non-glaucomatous healthy controls of Saudi origin were selected from the anterior segment and general ophthalmology clinic at King Abdulaziz University Hospital, Riyadh, Saudi Arabia as detailed elsewhere [7]. Briefly, clinical diagnosis of POAG patients (n = 188) were based on the presence of: (i) the disc or retinal nerve fiber layer (RNFL) defect; (ii) visual field abnormalities; and (iii) bilateral open angles on gonioscopy. Secondary glaucoma cases such as pigmentary glaucoma, uveitic, pseudoexfoliation, and history of steroid use or ocular trauma were excluded. Ethically-matched non-glaucomatous control subjects (n = 164) were: > 20 years of age at recruitment; normal IOP [< 21 mmHg without any anti-glaucoma medication]; open angles with normal optic disc on examination; and no history of ocular disease(s) or previous ophthalmic surgeries. Any subject refusing to participate in the study was excluded.

#### Genotyping of rs7081455 in *PLXDC2*

DNA samples were genotyped for rs7081455 using the TaqMan^®^ SNP Genotyping Assay (assay ID: C_29390339_10, Applied Biosystems Inc., Foster City, CA, USA) on ABI 7500 Real-Time PCR System (Applied Biosystems) using PCR conditions as described by the manufacturer. The details of the PCR reaction and cycling conditions have been described previously [7]. Genotype calling was done using the in-built automated 2-color allele discrimination software on ABI 7500.

#### Statistical analysis

All the statistical analyses were performed using SPSS version 22 (IBM Inc.’ Chicago, IIinois, USA). Pearson’s Chi square test was used to analyze for deviation from Hardy-Weinberg equilibrium (HWE), allelic, genotypic and model-based (additive, dominant and recessive) association with POAG as indicated. 2 × 2 table was used to calculate odds ratio (OR) and 95% confidence interval (CI). Mean differences between genotypes/groups were tested by independent sample t-test, one-way ANOVA and Kruskal–Wallis tests. In addition, effect of age, sex and genotype on the disease outcome was tested by binary logistic regression. A *p* value < 0.05 was considered statistically significant.

### Results

A total number of 352 subjects, consisting of 188 POAG cases and 164 controls, were genotyped for rs7081455 in this study. The demographic and other characteristics of subjects included in this study are shown in the Additional file 1: Table S1. Both the POAG patient and control groups were similar for age, gender distribution, smoking habit and other systemic co-morbidities such as diabetes, hypertension, hypercholesterolemia and heart disease status. However, there was trend towards higher individuals with in family history of glaucoma in POAG group as compared to controls (*p *= 0.053).

The genotype frequencies showed no significant deviation from HWE (*p *> 0.05). POAG patients and controls exhibited no significant genotype distribution under additive (Chi^2^ = 1.46, df = 2, *p *= 0.482), dominant (OR = 1.14, 95% CI 0.69–1.87, *p *= 0.590) and recessive models (OR = 1.32, 95% CI 0.87–2.07, *p *= 0.228). Besides, the allele frequency distribution was also non-significant (OR = 1.30; 95% CI 0.86–1.97; *p *= 0.197). The risk allele ‘G’ frequency was 0.56 and 0.52 in controls and patients, respectively (Table 1).

**Table 1:** Association analysis of allele frequency and genotype distribution for polymorphism rs7081455 in POAG cases and controls

SNP (gene)	rs7081455 (*PLXDC2*)				
	Controls (n = 164) No. (%)	POAG (n = 188) No. (%)	Odds ratio	95% confidence interval	*p* value^a^
Allelic analysis					
T	145 (44.0)	182 (48.0)	1	Reference	–
G*	183 (56.0)	194 (52.0)	1.30	0.86–1.97	0.197
HWE P	0.117	0.388	–	–	–
Genotype and model analysis					
TT	37 (22.5)	47 (25.0)	1	Reference	–
TG	71 (43.3)	88 (46.8)	1.02	0.60–1.74	0.920
GG	56 (34.1)	53 (28.2)	1.34	0.75–2.37	0.312
Additive	–	–	–	–	0.482
Dominant	–	–	1.14	0.69–1.87	0.590
Recessive	–	–	1.32	0.84–2.07	0.228

*HWE P* Hardy–Weinberg equilibrium *p* value

^a^Pearson Chi^2^ test

* Risk variant

The data was further analyzed for the effect of rs7081455 genotype on different demographic and clinical variables within the POAG patient group. There was no significant difference between age (p = 0.305) and gender (p = 0.894) distribution. Similarly, there was no significant genotype effect on family history of glaucoma, smoking and systemic diseases. More importantly, clinical phenotype traits such as IOP and cup/disc ratio; and number of anti-glaucoma medications, that also serve as markers to assess disease severity were also found to be statistically non-significant (Table 2). Besides, binary logistic regression analysis also showed no significant effect of age, sex and genotype on the likelihood of POAG occurrence.

**Table 2:** Genotype effect on demographic and clinical characteristics within PAOG group

Characteristics	Genotypes			*p* value^a^
	T/T (n = 47) No. (%)	T/G (n = 88) No. (%)	G/G (n = 53) No. (%)	
Demographic				
Age in years, mean (SD)	59.0 (11.3)	61.2 (11.0)	62.2 (9.4)	0.305*
Male	27 (57.4)	49 (55.6)	28 (52.8)	0.894
Female	20 (42.5)	39 (44.3)	25 (47.1)	–
Medical history				
Family history of glaucoma	5 (10.6)	7 (7.9)	6 (11.3)	0.773
Smoking	8 (17.0)	7 (7.9)	5 (9.4)	0.251
Diabetes mellitus	18 (38.3)	37 (42.0)	20 (37.7)	0.851
Hypertension	15 (31.9)	34 (38.6)	22 (41.5)	0.598
Coronary artery disease	1 (2.1)	3 (3.4)	2 (3.7)	0.885
Hypercholesterolemia	1 (2.1)	7 (7.9)	5 (9.4)	0.310
Glaucoma indices				
Intraocular pressure, mmHg, mean (SD)	22.3 (8.1)	23.7 (8.8)	23.2 (10.3)	0.536**
Cup/disc ratio	0.77 (0.2)	0.81 (0.6)	0.75 (0.2)	0.736**
Number of anti-glaucoma medications	1.6 (0.9)	1.9 (1.1)	1.8 (1.2)	0.214**

^a^Pearson Chi^2^ test

* One-way ANOVA

** Kruskal–Wallis test

### Discussion

Recent GWAS’s have identified numerous genetic variants in a number of genes/loci to be associated with POAG or its related clinical endophenotypes [4]. Using a similar genome-wide approach, Nakano et al. identified an intergenic SNP, rs7081455, near *PLXDC2* gene to be associated with POAG in the Japanese population [5]. This finding was subsequently replicated by Mabuchi et al. in POAG patients of same ethnicity and also demonstrated to be associated with increasing IOP. Since POAG is the second most prevalent type of glaucoma in Saudi Arabia [1], we investigated an association between SNP rs7081455 near *PLXDC2* gene and POAG or its endophenotype traits in a Saudi cohort.

Our study reports a negative association between rs7081455 and POAG or its glaucoma specific indices (endophenotypes) namely, IOP and cup/disc ratio. There have been conflicting reports on association between variant rs7081455 and POAG in the literature. Rs7081455 was associated with POAG in the Japanese population in two different reports [5, 6], but not by Takamoto et al. [8] in the same population in patients with normal tension glaucoma, substantiating an IOP-related effect of this variant on POAG as demonstrated by Mabuchi et al. [6]. Similarly, there was no association reported in the Chinese [9], Afro-Caribbean [10] and Korean [11] populations. Besides, among the two reports from India, there appeared to be a regional difference, with no association among the South Indian as compared to modest association among the POAG patients from Eastern parts of India [12, 13]. The distribution of the risk ‘G’ allele frequency observed in our study and other ethnic groups discussed above are shown in Table 3. The ‘G’ allele frequency of rs7081455 was highest in our Saudi cohort and lowest among the Chinese population.

**Table 3:** Frequency of risk variant ‘G’ allele of rs7081455 in different populations

Population	‘G’ allele frequency		*p* value	References
	Controls	POAG		
Japanese	0.17	0.26	0.00001	[5]
Japanese	0.18	0.24	0.005	[6]
Japanese	0.23	0.22	0.685	[8]
Chinese	0.13	0.15	0.310	[9]
Korean	0.20	0.21	0.740	[11]
Afro-Caribbean	0.30	0.29	0.805	[10]
South India	0.40	0.42	0.629	[12]
East India	0.33	0.38	0.048	[13]
Saudi	0.56	0.52	0.197	This Study

The PLXDC2 is cell surface trans-membrane protein receptor for pigment epithelium derived factor (PEDF) [14] that is widely expressed in many tissues including the eye and has been implicated to have a role in POAG [5, 6]. Though the exact mechanism(s) by which *PLXDC2* is involved in POAG development and/or progression is not yet clear, however, the genetic variant rs7081455 near *PLXDC2* seems to contribute to glaucomatous optic neuropathy in an IOP-related mechanism, because this locus was associated with high-tension glaucoma rather than normal-tension glaucoma [6]. The authors hypothesized that the variant may affect expression and/or activity of PLXDC2 protein leading to different level of responsiveness to PEDF, thereby causing elevated IOP [6]. Besides, Rogers et al. [15] has reported that PEDF decreases the conventional outflow pathway of the aqueous fluid by increasing the barrier function of the inner wall of Schlemm’s canal. Thus, this locus/variant may be associated with IOP-regulation by decreasing the outflow pathway via the PEDF-PLXDC2 receptor [6, 15]. In contrast, our study failed to observe this link as there was no significant association of this genotype with IOP or cup/disc ratio.

Validation or replication of association studies in different ethnic groups is of genetic epidemiological relevance to evaluate the future utility of genetic markers in disease per se. However, the findings observed in our study and those reported in other populations suggests that variant rs7081455 in *PLXDC2* may not have a major role to play in the pathogenesis of the disease and hence, may not be an important genetic marker that may serve as a useful tool to assess the risk of POAG.

## Limitations

Our study reports a negative association between rs7081455 and POAG. However, the lack of significance in our study could be due to relatively small sample size and insufficient statistical power of the study. Based on the minor allele frequency observed in our Saudi cohort, the current sample size of the study has an estimated power of > 80% (with alpha risk of 5%) to detect an odds ratio of 2.0. However, it will certainly require a much larger sample size to detect a 1.5-fold relative risk, as is the case with most genetic association studies where polymorphism(s) exhibit a relatively small effect but may show a significant contribution to a large number of cases, emphasizing the need for further validation in a large population-based cohort. In addition, only a single genetic variant in *PLXDC2* gene was studied, so the role of other rare variants in this gene or gene–gene interaction cannot be ruled out.

## Additional file

**Additional file 1: Table S1.** Demographic and clinical characteristics of POAG cases and controls genotyped for polymorphism rs7081455 included in this study.

## Abbreviations

HWE: Hardy–Weinberg equilibrium
IOP: intraocular pressure
PEDF: pigment epithelium derived factor
PLXDC2: plexin domain containing 2
POAG: primary open angle glaucoma
RFNL: retinal nerve fiber layer
