# Supplementary material for: Plexin domain containing 2 (PLXDC2) gene polymorphism rs7081455 may not influence POAG risk in a Saudi cohort
Source: BMC Res Notes. 2018 Oct 16;11:733. doi: 10.1186/s13104-018-3848-x (PMC6192173; doi:10.1186/s13104-018-3848-x)
Supplement: Supplementary file 1 — Additional file 1: Table S1. Demographic and clinical characteristics of POAG cases and controls genotyped for polymorphism rs7081455 included in this study. [file 13104_2018_3848_MOESM1_ESM.doc]

**Table S1.** Demographic and clinical characteristics of POAG cases and controls genotyped for polymorphism rs7081455 included in this study

| **Variables** | **Controls**  **(n = 164)**  **No. (%)** | **POAG**  **(n = 188)**  **No. (%)** | ***p* value^a^** |
| --- | --- | --- | --- |
| **Demographic Characteristics** |  |  |  |
| Age in years, mean (±SD) | 58.7 (11.4) | 60.9 (10.7) | 0.060* |
| Male | 94 (57.3) | 104 (55.3) | 0.706 |
| Female | 70 (42.7) | 84 (44.6) | - |
| **Systemic Diseases** |  |  |  |
| Diabetes mellitus | 62 (37.8) | 75 (39.9) | 0.688 |
| Coronary artery disease | 4 (2.4) | 6 (3.2) | 0.672 |
| Hypertension | 53 (32.3) | 71 (37.7) | 0.286 |
| Hypercholesterolemia | 8 (4.8) | 13 (6.9) | 0.421 |
| **Health Awareness/Behavior** |  |  |  |
| Family history of glaucoma | 7 (4.2) | 18 (9.5) | 0.053 |
| Smoking | 15 (9.1) | 20 (10.6) | 0.641 |

^a^Pearson Chi^2^ test, **t*-test
